# *Hypsizygus marmoreus* as a Source of Indole Compounds and Other Bioactive Substances with Health-Promoting Activities

**DOI:** 10.3390/molecules27248917

**Published:** 2022-12-15

**Authors:** Katarzyna Kała, Wojciech Pająk, Katarzyna Sułkowska-Ziaja, Agata Krakowska, Jan Lazur, Maciej Fidurski, Krystian Marzec, Piotr Zięba, Agata Fijałkowska, Agnieszka Szewczyk, Bożena Muszyńska

**Affiliations:** 1 Department of Pharmaceutical Botany, Faculty of Pharmacy, Jagiellonian University Medical College, 9 Medyczna, 30-688 Kraków, Poland; 2 Department of Inorganic and Analytical Chemistry, Faculty of Pharmacy, Jagiellonian University Medical College, 9 Medyczna, 30-688 Kraków, Poland; 3 Department of Horticulture, Faculty of Biotechnology and Horticulture, University of Agriculture in Kraków, 54 29-Listopada, 31-425 Kraków, Poland

**Keywords:** shimeji mushrooms, indole compounds, lovastatin, ergothioneine, glucans, phenolic compounds, antioxidant activity

## Abstract

*Hypsizygus marmoreus* is an edible medicinal mushroom species with a high dietary value. In this study, the fruiting bodies of commercial and self-cultivated crops and mycelium from in vitro *H. marmoreus* cultures (both white and brown varieties) were evaluated. This study aimed to analyze the presence of indole compounds and other biologically active substances and determine the effect that the addition of zinc and magnesium ions to the culture medium has on the content of the tested compounds in mycelial cultures. The content of indole compounds and other organic compounds was determined using high-performance liquid chromatography, the content of bioelements was determined using flame atomic absorption spectrometry, the glucan content was determined spectrophotometrically, and the antioxidant activity of extracts was estimated using DPPH, FRAP, and ABTS methods. The results showed that *H. marmoreus* mycelium from in vitro cultures is a good source of indole compounds, bioelements, glucans, and lovastatin. Mycelia from in vitro cultures showed the most diverse composition of indole compounds (L-tryptophan, 5-hydroxy-L-tryptophan, tryptamine, 5-methyltryptamine, and melatonin). Additionally, in vitro cultures of *H. marmoreus* enriched with Zn and Mg salts increased the contents of Na, Ca, Zn, 5-methyltryptamine, melatonin, protocatechuic acid, sterols, and total glucans. Only in the case of the white variety of mycelial enriched cultures, ergothioneine and Mg levels also increased. To summarize, the content of the active compounds differed depending on the *H. marmoreus* variety and the tested material.

## 1. Introduction

The nutritional and health-promoting properties of mushrooms were reported thousands of years ago, and mushroom cultivation in Asian countries, particularly in China, dates back several centuries [1]. The anti-inflammatory, immunostimulating, and anticancer effects of various types of extracts obtained from mushrooms have been known since ancient times in China, Japan, and other eastern countries [2,3,4].

In recent years, the antioxidant compounds found in mushrooms have gained immense attention as they are reported to have a positive effect in humans by, among other things, protecting DNA from oxidative changes [2,4,5]. Indole compounds are one of the most important antioxidant compounds found in many mushroom species, and they determine the dietary and medicinal value of mushrooms. These compounds play an extremely important role in the prevention of depression, besides showing procognitive and antioxidant activities. Other compounds were also identified in mushrooms, such as ergothioneine, which is a thiol compound with strong antioxidant activity, sterols, lovastatin, phenolic compounds, glucans, and macro- and microelements [3,5,6].

The nutritional and medicinal properties of *Hypsizygus marmoreus* (Peck) H.E. Bigelow (Shimeji) are well established. Its fruiting bodies have a unique sweet and nutty flavor and a crunchy texture, which make this species increasingly popular worldwide for culinary purposes [7]. The natural habitats of this species are Korea, Japan, China, and northern Europe, and they grow primarily in autumn and winter. Japan is the largest producer and consumer of this species, and its commercial cultivation there began in 1972 [8]. Two varieties of this species are grown for commercial use: white and brown. The currently available studies, mainly from Asian countries, focus on the therapeutic effects of *H. marmoreus*. Its dietary effect is attributed to active substances such as polysaccharides, proteins, essential amino acids, lectins, vitamins, and enzymes [7]. Due to the presence of these compounds, this species shows, among other things, anti-inflammatory, antioxidant, antihypertensive, anticancer, and antiallergic properties [9,10].

This study aimed to determine, both qualitatively and quantitatively, the biologically active compounds and bioelements in white and brown *H. marmoreus* fruiting bodies obtained from commercial and self-cultivation and in mycelial cultures of *H. marmoreus* of both varieties. In the experiment conducted, in vitro cultures of *H. marmoreus* were established and studied. This is important because the available scientific data are mainly based on material sourced from Asia [7,8,9,10]. Using in vitro cultures, a high biomass of the species can be achieved in a relatively short time [11,12]. Furthermore, modifying the composition of the substrate can affect the production of active metabolites. Due to their ability to absorb bioelements from the substrate, mycelia from in vitro cultures can be successfully enriched with bioelements. Moreover, the enrichment of substrates with the precursors of bioactive substances, such as phenylalanine, can significantly affect the mushrooms’ metabolism by enhancing the synthesis of phenolic acids with antioxidant activity [11,13]. Another of the methods of enriching mycelial cultures with bioactive compounds is elicitation, which affects the synthesis pathway of secondary metabolites, causing their increased synthesis or accumulation [14,15]. Thus, this study also aimed to determine the effect of the addition of zinc and magnesium salts to the culture media on the content of bioelements and other active substances in *H. marmoreus*.

The plan for the experiment included obtaining mycelial cultures and fruiting bodies from both self-cultivated and commercially sourced *H. marmoreus* species of two varieties, brown and white. Mycelial cultures were also obtained on media enriched in Zn and Mg to verify how the addition of these elements would affect the content of the analyzed substances. The material thus prepared was lyophilized and extracted to determine the content of bioactive compounds, as well as the antioxidant potential. The content of bioactive compounds was tested using high-performance liquid chromatography (HPLC), the content of bioelements was determined using flame atomic absorption spectrometry (FAAS), the glucan content was determined spectrophotometrically, and the antioxidant activity of these compounds was estimated using the DPPH, FRAP, and ABTS methods. The results obtained were then analyzed using statistical tools.

This research is particularly important because the biologically active substances present in *H. marmoreus* have not yet been extensively studied. This emphasizes the importance of the qualitative and quantitative analyses of compounds such as indole, phenolic compounds, lovastatin, ergothioneine, phenylalanine, bioelements, sterols, and glucans carried out in this study. In addition, the antioxidant potential of *H. marmoreus* fruiting bodies and mycelial cultures was investigated. Along with the brown variety of *H. marmoreus*, which has been analyzed in many studies, the less common white variety was also analyzed.

## 2. Results

### 2.1. Analysis of Bioelements

The content of the essential bioelements in the samples is presented in Table 1.

In the analysis of the bioelement content of the *H. marmoreus* fruiting bodies from both own cultivation and commercial origins, as well as of nonenriched mycelial cultures, the brown variety was found to be a better source of the bioelements determined in each case. Among the analyzed fruiting bodies, the best source of Mg, Zn, Cu, and Fe was the brown variety of commercial origin, while for Na, K, and Ca the best source was the brown variety obtained from own cultivation. In the analysis of mycelial cultures enriched with Mg and Zn salts, the white variety was found to be a better source of Ca, Cu, and Fe, whereas the brown variety showed a higher content of Na, K, Mg, and Zn. After the enrichment of the mycelium, the Mg and Zn content was 330 and 30.2 mg 100 g^−1^ dry weight (d.w.) in the brown variety mycelium and 303 and 29.1 mg 100 g^−1^ d.w. in the white variety mycelium, respectively. The addition of zinc and magnesium salts to the medium significantly increased the zinc content and significantly decreased the copper content compared with the nonenriched cultures (Table 1). Furthermore, the enrichment of the medium with Zn and Mg resulted in an approximately twofold increase in the Zn content in the mycelium but only a few percentages of increase in the Mg content. The increase in Zn and Mg content was nearly 74% and 1.5% for the brown variety and 127% and 6% for the white variety.

### 2.2. Analysis of Indole Compounds

The results of the analysis of indole compounds in the *H. marmoreus* samples are presented in Table 2.

Analysis of the indole compounds revealed the presence of six indole compounds: L-tryptophan, 5-hydroxy-L-tryptophan, serotonin, tryptamine, 5-methyltryptamine, and melatonin. In the analysis, L-tryptophan, which is a key essential amino acid and also a precursor of neurotransmitters in the body, was detected in all samples, and its highest content was observed in the brown *H. marmoreus* fruiting bodies obtained commercially [16,17]. The L-tryptophan content ranged from 8.97 mg 100 g^−1^ d.w. (in the brown variety fruiting bodies obtained from own cultivation) to 72.1 mg 100 g^−1^ d.w. (in the brown variety fruiting bodies of commercial origin). In mycelial cultures, the L-tryptophan content ranged from 17.3 to 25.6 mg 100 g^−1^ d.w. (Table 2). Serotonin was not determined quantitatively in any of the extracts tested. Trace amounts of serotonin were detected in the brown variety of commercial origin and the mycelium of the white variety grown on the medium enriched with zinc and magnesium salts. Melatonin was detected in six of the eight samples tested, of which the quantitative analysis was carried out only using the white and brown mycelium from the Mg- and Zn-enriched cultures. The melatonin content was found to be 4.37 and 4.99 mg 100 g^−1^ d.w. in the white and brown varieties, respectively. The highest content of tryptamine and 5-methyltryptamine was observed in the commercially obtained brown variety fruiting bodies, at 29.3 and 27.2 mg 100 g^−1^ d.w., respectively. The presence of 5-HTP was detected only in three extracts, with the highest content being found in the mycelial cultures of the brown variety—11.4 mg 100 g^−1^ d.w. It is worth noting that the mycelial cultures showed more diverse forms of indole compounds, whereas the *H. marmoreus* fruiting bodies may be a potential source of only L-tryptophan, tryptamine, and 5-methyltryptamine (Table 2).

### 2.3. Analysis of Sterol Compounds

The content of ergosterol and ergosterol peroxide in the analyzed *H. marmoreus* samples is presented in Table 2. Among the analyzed *H. marmoreus* samples, the highest ergosterol content was observed in the brown mycelium grown on the enriched medium (166 mg 100 g^−1^ d.w.). A high ergosterol content was also observed in the white mycelium (enriched cultures) and in the brown fruiting bodies of commercial origin (142 and 116 mg 100 g^−1^ d.w., respectively). Ergosterol peroxide was quantified in four of the samples tested: in the brown and white mycelium grown on the enriched medium and in the white and brown fruiting bodies of commercial origin. The highest ergosterol peroxide content (15.8 mg 100 g^−1^ d.w.) was observed in the brown mycelium (enriched mycelial cultures). Ergosterol peroxide was not detected in the white fruiting bodies obtained from our own crops. The remaining samples showed the presence of ergosterol peroxide only qualitatively. The addition of magnesium and zinc salts to the culture medium increased both the ergosterol and ergosterol peroxide contents in the mycelium compared with the nonenriched cultures (Table 2).

### 2.4. Analysis of Lovastatin Content

The lovastatin content of the analyzed samples is presented in Table 2. Among the analyzed *H. marmoreus* extracts, the highest lovastatin content was observed in the white fruiting bodies of commercial origin and the brown fruiting bodies obtained from own cultivation (74.5 and 66.7 mg 100 g^−1^ d.w., respectively). The lowest lovastatin content was observed in the in vitro mycelium of the brown variety grown on the enriched medium (15.0 mg 100 g^−1^ d.w.). In both the white and the brown varieties, a higher lovastatin content was observed in the fruiting bodies than in the mycelium, regardless of the type of substrate on which they were grown. These findings demonstrated that the addition of zinc and magnesium salts to the culture medium results in a reduction in the lovastatin content compared with the nonenriched culture, which is particularly noticeable in the case of the white variety (a reduction of nearly 50% in the lovastatin content) (Table 2).

### 2.5. Analysis of Ergothioneine Content

Among the *H. marmoreus* extracts tested, the highest ergothioneine content was determined in the mycelium of the white variety grown on the enriched medium (80.4 mg 100 g^−1^ d.w.). Brown mycelial cultures grown on the control medium also showed a significant ergothioneine content (73.0 mg 100 g^−1^ d.w.). In the remaining samples, the ergothioneine content ranged from 14.7 to 25.6 mg 100 g^−1^ d.w., which may indicate that *H. marmoreus* is a potential source of this antioxidant (Table 2).

### 2.6. Analysis of Phenolic Compounds and Phenylalanine Content

Among the analyzed samples, the highest phenylalanine and cinnamic acid contents were determined in the brown fruiting bodies of commercial origin (422 and 8.88 mg 100 g^−1^ d.w., respectively, Table 2). Vanillic acid was detected only in the white fruiting bodies of commercial origin, with a content of 4.21 mg 100 g^−1^ d.w. These fruiting bodies also showed a significant content of L-phenylalanine (292 mg 100 g^−1^ d.w.). The highest contents of protocatechuic acid and p-hydroxybenzoic acid (16.6 and 23.5 mg 100 g^−1^ d.w., respectively) were also observed in these fruiting bodies. Protocatechuic acid and p-hydroxybenzoic acid were determined in the majority of the analyzed samples. In the mycelium from the in vitro cultures, the addition of zinc and magnesium salts to the culture medium resulted in a reduction in the phenylalanine content compared with the nonenriched cultures. The L-phenylalanine content decreased particularly in the mycelial cultures of the brown variety (by more than 50%) (Table 2).

### 2.7. Analysis of Glucan Content

As shown in Table 2, the highest glucan content was observed in the white and brown fruiting bodies obtained from own cultivation (61.4 and 58.0 g 100 g^−1^ d.w., respectively), which may indicate the commercial potential of such cultivation. These samples also showed the highest β-glucan content. The lowest glucan content was observed in the brown fruiting bodies of commercial origin. In mycelial cultures, the addition of Zn and Mg salts resulted in an increase in the content of both total glucans and β-glucans compared with the mycelia from in vitro cultures grown on the control medium (Table 2).

### 2.8. Analysis of Antioxidant Potential

**Figure 1:**
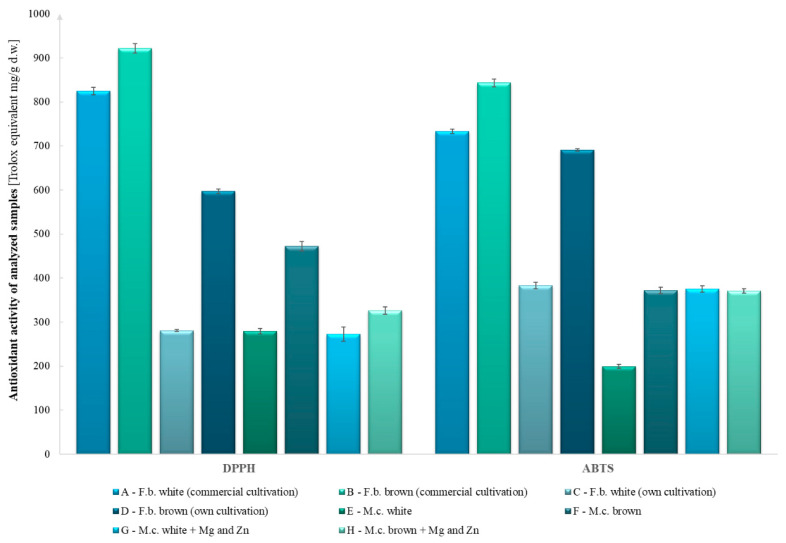
Comparison of antioxidant activity by DPPH and ABTS methods.

The highest antioxidant activity using the DPPH method was observed in the brown fruiting bodies of commercial origin, closely followed by the white fruiting bodies of commercial origin (922 and 825 mg TE g^−1^ d.w., respectively). The lowest antioxidant potential was observed in the enriched and control white mycelial cultures (273 and 279 mg TE g^−1^ d.w., respectively). Detailed results are presented in Figure 1.

**Figure 2:**
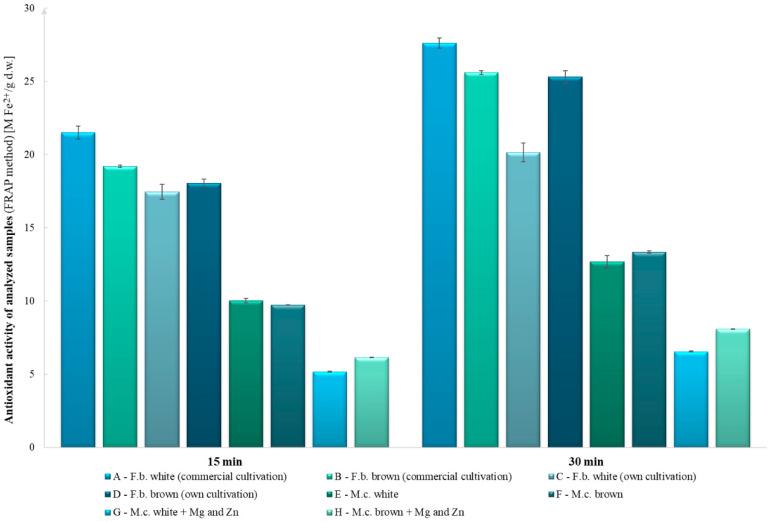
Comparison of antioxidant activity using the FRAP method (after 15 and 30 min).

The highest antioxidant activity using the ABTS method was observed in the white and brown fruiting bodies of commercial origin (843 and 733 mg TE g^−1^ d.w., respectively). The lowest antioxidant activity was observed in the white mycelial cultures and the enriched brown mycelial cultures (200 and 371 mg TE g^−1^ d.w., respectively, Figure 1). Based on the results of the FRAP test, the highest antioxidant potential was observed in the mushrooms of commercial origin and the fruiting bodies obtained from own cultivation, whereas the lowest antioxidant potential was observed in the enriched mycelial cultures modified with zinc and magnesium (Figure 2).

In all tests conducted, the addition of zinc and magnesium compounds to the substrate reduced the antioxidant activity compared with the control objects, except for in the ABTS test of the enriched white mycelial in vitro cultures. The differences between the results of ABTS and DPPH assays may be due to the different reaction mechanisms and also to structural differences in the biologically active compounds. The ABTS method has the advantage of being able to analyze compounds at different pH levels, whereas the DPPH method does not. Thus, the ABTS method can be used to evaluate the effects of an acidic pH on antioxidant activity. It is also useful in extracting compounds from acidic solvents and evaluating the antioxidant activity of various media. In addition, it can be used in different media, such as organic and aqueous solutions [18]. It can be presumed that the differences in the results obtained between the fruiting bodies and the mycelial cultures are due to the functions of the mushrooms at a given stage (Figure 1 and Figure 2). In the early stages, such as the formation of mycelium, more minerals and also growth-promoting materials are needed to form the fruiting bodies, which store substances needed for survival and biochemical assays.

## 3. Discussion

The brown and white varieties of *H. marmoreus* (family *Lyophyllaceae*) were analyzed. This species is one of the most popular mushrooms in Asia, particularly in Japan, which is its highest producer. The majority of *H. marmoreus* are sold as food products, but increasing attention has also been focused on their medicinal properties. Studies have reported the anticancer, anti-inflammatory, antifungal, and antioxidant activities of *H. marmoreus* extracts [8]. In addition, it is worth noting that the potential of edible mushrooms, including the species *H. marmoreus*, is multidirectional, with a particular focus on their use as functional foods and even potential dietary supplements and medicines [4,7,9,19].

First, the content of selected bioelements was analyzed, followed by macroelements (Na, K, Ca, and Mg) and microelements (Zn, Fe, and Cu). Bioelements are essential for the proper functioning of the human body, and their deficiency can lead to various disorders. These deficiencies can be prevented by enriching functional foods with bioelements [11]. The ability of mushrooms to accumulate bioelements is attributable to several factors, the most important being species characteristics related to nutrient uptake dynamics and the composition of the culture medium. Saprotrophic mushrooms such as *H. marmoreus* take up organic matter osmotropically, i.e., through the accumulation of bioelements in the hyphae [11]. Analyses revealed that *H. marmoreus* is a good source of potassium. The highest potassium content was observed in the brown fruiting bodies obtained from own cultivation (2656 mg 100 g^−1^ d.w.), which is more than twice as high as, for example, in dried apricots, which are considered a good source of potassium [20]. In the remaining samples, the potassium content ranged from 564 to 2159 mg 100 g^−1^ d.w. The extracts analyzed were characterized by low sodium and calcium contents. The highest calcium content was observed in the mycelia grown on the medium enriched with zinc and magnesium salts (25.8 and 25.7 mg 100 g^−1^ d.w. in the white and brown varieties, respectively), which are almost six times less than the values reported by Karmanska [21]. The magnesium content in the samples ranged from 197 mg 100 g^−1^ d.w. in the white fruiting bodies obtained from self-cultivation to 330 mg 100 g^−1^ d.w. in the mycelium of the brown variety grown on the enriched cultures, which are higher than the values reported by Karmanska [21]. The highest iron and copper contents were observed in the mycelia of the brown variety (9.08 and 3.53 mg 100 g^−1^ d.w., respectively). These values are significantly different from the values reported by Karmanska, who reported an iron content of 19.0 mg 100 g^−1^ d.w. and a copper content of 1.32 mg 100 g^−1^ d.w. [21]. These differences may be due to the use of different culture media. In the present study, the addition of magnesium and zinc salts to the medium resulted in a significant increase in the calcium, sodium, and zinc contents in the mycelia, compared with the nonenriched culture, and did not affect the potassium and magnesium contents. The highest increase, almost double, was observed in the zinc content. In contrast, the addition of magnesium and zinc salts to the medium reduced the copper content. Similar results were reported for *Pleurotus* spp. by Włodarczyk et al., who—following the addition of zinc ions in the form of zinc sulfate to the medium—observed a significant increase in the zinc content in mycelial cultures of *Pleurotus* spp. compared with nonenriched cultures [22].

It should be emphasized that this is the first study to report the presence in *H. marmoreus* of indole compounds, which are an extremely important group of compounds with procognitive, antidepressant, antioxidant, and neuroprotective properties [16,17]. L-Tryptophan was detected in all of the samples analyzed, and its highest content was observed in the brown fruiting bodies of commercial origin. The L-tryptophan content was significantly higher in both the fruiting bodies and in vitro mycelial cultures of *H. marmoreus* compared with another commercially sourced species from China, *Cordyceps militaris*, whose L-tryptophan content ranged from 5.84 in the mycelium to 8.71 mg 100 g^−1^ d.w. in the fruiting bodies [12]. Serotonin was not quantified in any of the extracts analyzed, indicating variability among different edible mushroom species [16]. It was detected in trace amounts in two of the eight samples analyzed (Table 2). Melatonin was detected in six extracts, but only the white and brown mycelia from the enriched culture were quantitatively analyzed. This finding is particularly important because melatonin is a natural antioxidant and also has anticancer and antiaging activities, with roles in coordinating the daytime rhythm and neuroprotective function [16]. It is also present in other edible mushroom species in trace amounts, though this was determined after extraction under artificial digestive tract conditions in some cases [12,16,23]. The melatonin content in the white and brown mycelia from the enriched culture was 4.37 and 4.99 mg 100 g^−1^ d.w., respectively (Table 2). The highest tryptamine and 5-methyltryptamine contents were observed in the brown fruiting bodies of commercial origin. These compounds have also been determined in other edible mushroom species, and their health-promoting effects and potential to undergo digestive processes have been reported [12,16,23]. The compound 5-HTP is a direct serotonin precursor. When consumed with food, it easily penetrates the blood–brain barrier in the central nervous system, where it is transformed into serotonin. It has antidepressant and sleeping effects and is also used as an appetite suppressant and in the treatment of anxiety [17]. In this study, 5-HTP was observed in only three samples from mycelial cultures, indicating that mycelium from in vitro cultures is a better source of this compound than fruiting bodies. Previous studies have confirmed that mycelial cultures can be a source of this antidepressant [24]. In addition, it is reported that mycelial cultures grown on control Oddoux media are a better source of this compound [24]. However, no effect of medium modification on the L-tryptophan content was observed (Table 2). It is important to emphasize that 5-HTP and other indole-derived compounds are the subject of research not only in animal models but also in numerous human clinical trials [17].

Sterols, especially ergosterol, are converted into vitamin D_2_ in the presence of ultraviolet rays, which plays an important role in the prevention of cancer and regulates other immunomodulatory functions. Ergosterol is also responsible for the absorption of Ca and P, which contribute to the proper functioning of the skeletal system [25]. The highest ergosterol content was observed in the brown mycelium grown on the enriched medium (166 mg 100 g^−1^ d.w.). The ergosterol content in the remaining samples ranged from 24.9 to 142 mg 100 g^−1^ d.w. These results are consistent with those obtained by Jang et al., who reported an ergosterol content of 48–121 mg 100 g^−1^ d.w. in *H. marmoreus* [26]. Ergosterol peroxide, a metabolite of ergosterol, was quantified in only four of the eight extracts analyzed, with the highest levels being observed in the brown mycelium grown on the enriched culture. The addition of zinc and magnesium ions to the culture medium resulted in an increase in the content of both compounds in the mycelial cultures. A nearly sevenfold increase and a nearly threefold increase in the ergosterol content were observed for the white and brown varieties, respectively. The enrichment of the media resulted in an increase in the ergosterol peroxide content, enabling its quantitative analysis. In the white mycelium, 6.01 mg 100 g^−1^ d.w. of ergosterol peroxide was observed, and in the brown cultivar, it was 15.8 mg 100 g^−1^ d.w., which is similar to the values reported for *Cortinarius caperatus* (14.3 mg 100 g^−1^ d.w.) and *Leccinum scabrum* (14.9 mg 100 g^−1^ d.w.), but much lower than those for other edible species such as *Imleria badia* (87.6 mg 100 g^−1^ d.w.) and *Laetiporus sulphureus* (193 mg 100 g^−1^ d.w.) [27].

In addition to its typical use in the treatment of hypercholesterolemia, lovastatin also has neuroprotective, antidepressant, anti-inflammatory, and antitumor effects [28]. The lovastatin content in the fruiting bodies of several mushrooms has been investigated by Lo et al., among others [29]. Nineteen species of the genera *Agrocybe*, *Agaricus*, *Boletus*, *Ganoderma*, *Inonotus*, *Lentinus*, *Pleurotus*, *Morchella*, *Volvariella*, *Auricularia*, and *Termitomyces*, including *H. marmoreus*, were extensively analyzed [29]. In their study, the lovastatin content in the fruiting bodies of the brown variety of *H. marmoreus* was found to be 62.8 mg 100 g^−1^ d.w., which was the highest of all the mushrooms analyzed. This result is consistent with the finding of the present study, in which a lovastatin content of 62.4 and 66.7 mg 100 g^−1^ d.w. was reported for the brown fruiting bodies of commercial origin and the brown fruiting bodies obtained from own cultivation, respectively. However, the highest lovastatin content was observed in the white fruiting bodies of commercial origin. The addition of zinc and magnesium to the medium reduced the lovastatin content in mycelial cultures. A twofold decrease in the lovastatin content was observed in the white variety compared with the nonenriched culture (Table 2). Zięba et al. reported a similar finding in their research on *Pleurotus eryngii* mycelium [13]. The enrichment of the medium with zinc ions in the form of zinc sulfate or zinc hydrogenaspartate resulted in a drastic decrease in the lovastatin content [13].

The highest ergothioneine (a powerful antioxidant with photoprotective activity) content was observed in the brown mycelium (73.0 mg 100 g^−1^ d.w.) [30]. In the remaining extracts, the ergothioneine content ranged from 14.65 to 40.21 mg 100 g^−1^ d.w. In comparison, Kalaras et al. reported an ergothioneine content of 727 mg 100 g^−1^ d.w. in *Boletus edulis* fruiting bodies, 394 mg 100 g^−1^ d.w. in *Pleurotus citrinopileatus* fruiting bodies, and 41 mg 100 g^−1^ d.w. in *Agaricus bisporus* fruiting bodies [31]. The most similar ergothioneine content was observed in *Cantharellus cibarius*. Lo et al. analyzed the ergothioneine content in the brown variety of *H. marmoreus* [29]. They reported an ergothioneine content of 4.6 mg 100 g^−1^ d.w., which is about five times lower than that determined using a similar sample in the present study [29]. Furthermore, the addition of zinc and magnesium salts to the culture medium resulted in an increase in the ergothioneine content in the white mycelium but a decrease in the brown mycelium, indicating the differences between the two varieties.

Phenolic compounds are included among in the health-promoting components due to their antioxidant activity, which results in their significant therapeutic potential [11]. In the present study, in addition to L-phenylalanine, the following phenolic compounds were detected in the *H. marmoreus* samples: protocatechuic acid, p-hydroxybenzoic acid, vanillic acid, and cinnamic acid. To date, few studies have reported the presence of these compounds in *H. marmoreus*. Xu et al. determined the total phenolic compounds in both varieties of *H. marmoreus* and identified three substances: catechin, gallic acid, and protocatechuic acid [10]. In the present study, the highest protocatechuic acid content was observed in the white fruiting bodies of commercial origin (16.6 mg 100 g^−1^ d.w.). Comparison of this value with the value reported by Xu et al. is difficult as they expressed their results as per unit of fresh weight. However, based on the analysis of the results of Xu et al., it can be concluded that the brown fruiting bodies are a better source of phenolic compounds, including protocatechuic acid [10]. This finding is in contrast to that of the present study, where the content of phenolic compounds was higher in the white fruiting bodies than in the brown fruiting bodies. The remaining phenolic compounds, i.e., p-hydroxybenzoic acid and vanillic acid, were determined for the first time in this species in the present study (Table 2). The addition of zinc and magnesium salts to the culture medium showed a negative effect on the L-phenylalanine content in the mycelium compared with the nonenriched culture, which may be comparable with the results of previous studies (Table 2) [11].

The glucan content in *H. marmoreus* has not been a frequent topic of research so far. In the present study, the total glucan content and also α-glucans and β-glucans were determined (Table 2). The highest glucan content was observed in the self-cultivated white fruiting bodies. The concentrations of total glucans, α-glucans, and β-glucans were 61.4 g 100 g^−1^ d.w., 13.2 g 100 g^−1^ d.w., and 48.2 g 100 g^−1^ d.w., respectively. Mirończuk-Chodakowska et al. analyzed the glucan content of popular mushrooms in Poland [32]. They evaluated popular commercially obtained mushrooms, including *Lentinula edodes* (total glucan content 24.7–30.4 g 100 g^−1^ d.w.; α-glucans 0.38–0.47 g 100 g^−1^ d.w.; β-glucans 24.7–30.4 g 100 g^−1^ d.w.), *Pleurotus ostreatus* (total glucan content 45.0–47.8 g 100 g^−1^ d.w.; α-glucans 5.09–6.29 g 100 g^−1^ d.w.; β-glucans 39.6–41.5 g 100 g^−1^ d.w.), and *A. bisporus* (total glucans 12.6–19.5 g 100 g^−1^ d.w.; α-glucans 2.41–6.48 g 100 g^−1^ d.w.; β-glucans 8.07–13.2 g 100 g^−1^ d.w.). Interestingly, they also reported the following contents of total glucans, α-glucans, and β-glucans in *H. marmoreus*: 32.0–61.3, 1.93–13.2, and 23.4–48.2 g 100 g^−1^ d.w., respectively. These results can be considered similar to those obtained in the present study [32]. Based on these data, it can be concluded that *H. marmoreus* is a competitive source of glucans.

The study of the antioxidant activity of *H. marmoreus* also proved to be extremely valuable as this aspect of the species has not been widely researched. Antunes et al. reported that commercially cultivated *L. edodes* has a higher antioxidant activity than *H. marmoreus* [33]. In addition, a lower total phenolic compound content is observed in shimeji compared with other mushroom species, which may translate into the antioxidant activity studied [33]. Other studies have confirmed that *H. marmoreus* fruiting bodies show a higher antioxidant activity than mycelia from in vitro cultures, which was also confirmed in the present experiment [34]. These compounds can act as proton donors or proton acceptors depending on the content of different compounds present in the extracts and different mechanisms of free radical scavenging. In general, a higher content of polyphenols, L-ascorbic acid, and other biologically active groups is positively correlated with a higher antioxidant activity [35]. Not only in the case of mushrooms, but also in other foods (e.g., olive oil), a correlation between the content of phenolic compounds and the antioxidant potential has been observed [36]. An important consideration when testing antioxidant potential is whether fresh or stored mushrooms were tested. In previous studies, it was proven that, for *Tuber melanosporum* species, the highest antioxidant potential was shown by fresh fruiting bodies, which is important for consumers, and the same is also possible for *H. marmoreus* [37].

## 4. Materials and Methods

### 4.1. Mushroom Material and Biotechnological Methods

**Figure 3:**
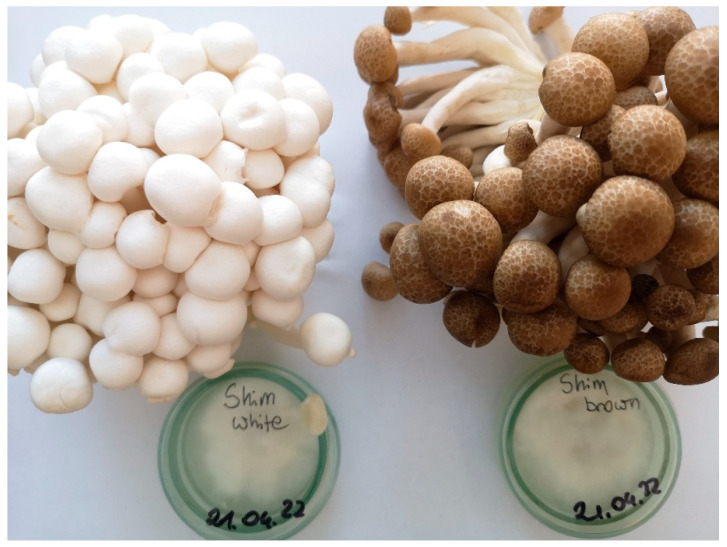
Fruiting bodies and mycelia on a solid medium of *H. marmoreus* white and brown varieties (photo by Katarzyna Kała).

Mycelia from in vitro cultures and fruiting bodies of the white and brown varieties of *H. marmoreus* (Peck) H.E. Bigelow of self-cultivation and commercial origins were used in this study (Figure 3). The samples of each variety were deposited in the Department of Pharmaceutical Botany, Jagiellonian University Medical College.

#### 4.1.1. Mycelial Cultures on a Solid Medium

In vitro stationary cultures were derived using hymenophore fragments of *H. marmoreus* fruiting bodies of commercial origin. The hymenium was degreased using 70° ethanol and then sterilized in a 15% sodium chlorate solution for 1 min. Then, the fruiting body fragments were rinsed with redistilled water, transferred to a Petri dish with a solid medium, following the procedure of Oddoux, and protected with parafilm [38]. The transfer of the fruiting bodies was performed in a laminar-ventilated cabinet.

#### 4.1.2. Shaken Cultures

Shaken cultures on a liquid medium were derived from the mycelium from the in vitro cultures on the solid medium. Mycelial fragments were obtained from the solid cultures in a laminar flow cabinet and transferred to 500-mL Erlenmeyer flasks containing 250 mL of the medium. The flasks were protected with aluminum foil and parafilm. The resulting cultures were shaken at 140 rpm for 2 weeks in a TOS-6048FD orbital motion rotary shaker (EnviSense, Lublin, Poland).

#### 4.1.3. Aerated Cultures in a Bioreactor

Two weeks after the liquid cultures were established, the biomass was separated from the substrate, rinsed with redistilled water, and transferred to two 10-L bioreactors. One bioreactor was filled with the liquid Oddoux medium and the other with the same medium but with zinc and magnesium salts added. Bioelements salts were added in powder form at the stage of preparing the liquid medium according to Oddoux. The salts of these bioelements were added such that 20 mg L^−1^ and 400 mg L^−1^ concentrations of zinc and magnesium ions were achieved in the medium, respectively. The cultures were carried out in the bioreactors for 10 days with continuous mixing using an air-lift system. The air introduced into the bioreactor was sterilized using an antimicrobial filter (Millex-61 GV Syringe Filter Unit, 0.22 µm, PVDF, 33 mm), and the mixing was ensured by a bubble flow with carbon dioxide produced by the mycelium. After growth, the mycelium was separated from the medium, washed with quadruple distilled water, frozen, and freeze-dried at −40 °C (Labconco Freezone lyophilizer 4.5, Kansas City, MO, USA). The cultures were conducted in the bioreactors to obtain sufficient biomass for further mycochemical analyses.

#### 4.1.4. Obtaining Fruiting Bodies of *H. marmoreus* in Self-Cultivation

Cultivation cultures on natural media were prepared using mycelium isolated in an earlier stage of the experiment. Agar cultures were inoculated into sterilized and rehydrated wheat grains previously stored in polypropylene bags with microfilters (Unicorn Bags, Forest, VA, USA). The bags were incubated at 22 ± 2 °C without access to light until the mycelium was fully overgrown—up to 3 weeks. Then, a growing medium of beech pellets and wheat bran in a 5:2 ratio was prepared. The dry substrate was rehydrated to a relative humidity of 65% and transferred to microfilter bags with 2 kg of substrate per experimental bag. Then, the bags containing the substrate and grains were subjected to steam sterilization for 2 h at 121 °C and 1 atm. After the substrate was cooled, it was inoculated with the previously prepared grained mycelium, i.e., 3% mycelium per 2 kg bag, mixed with grains, and stored in the incubation room according to the conditions for the grained mycelium. After full overgrowth, the substrate was cooled to 10 °C and stored in a growing chamber under the following conditions: constant air exchange, temperature of 15 °C, 95% humidity, and lighting of 600 lux. Fruiting bodies of the mushrooms were harvested after 3 weeks at commercial maturity, frozen, lyophilized (Labconco Freezone lyophilizer 4.5, Kansas City, MO, USA) at −40 °C, and used for further analyses.

### 4.2. Bioelement Analysis

#### 4.2.1. Mineralization of Samples

The study followed the procedure of wet mineralization using the Magnum II (ERTEC—Poland, Wrocław, Poland) microwave apparatus. The samples for mineralization were prepared using 0.2 g of dried fruiting bodies of commercial and self-cultured origins and mycelium obtained from in vitro cultures. The material was weighed, transferred to an agate mortar, ground to powder, and then stored in Teflon vessels. The mineralization process consisted of three 10-min steps and was carried out at 290 °C. The resulting solutions were transferred to quartz evaporators and evaporated at 150 °C using a hotplate. The residue obtained after the mineralization process was transferred to 10-mL volumetric flasks filled with quadruple distilled water.

#### 4.2.2. Quantitative Analysis

Bioelements in the samples were quantified using an iCE3500 spectrometer (Thermo Scientific, Gloucester, UK), followed by the FAAS method. The contents of the selected microelements (Mn, Fe, Zn, and Cu) and macroelements (Mg, K, Na, and Ca) were determined. Each sample was analyzed in nine independent replicates. The content of each bioelement in the samples was expressed as mg 100 g^−1^ d.w. ± standard deviation (SD).

### 4.3. Analysis of the Content of Organic Compounds

#### 4.3.1. Preparation of Methanolic Extracts

The mushroom material obtained was homogenized using an agate mortar. Of each sample, 4 g were transferred to 150-mL glass beakers, and 125 mL of analytical-grade methanol was added to each beaker. Extraction was carried out using ultrasound. For this purpose, the beakers with the biomass and methanol were kept in an ultrasonic bath (Polsonic, Warszawa, Poland) for 20 min. Then, the extracts were poured through fluted filters into 300-mL crystallizers and left at room temperature for the evaporation of methanol. This process was repeated six times. After the complete evaporation of methanol from the crystallizers, the dry residue was dissolved in HPLC-grade methanol and filtered using membrane filters into plastic tubes. The resulting extracts were stored in a refrigerator. For the chromatographic analyses, 2 mL of each extract was taken and filtered using 0.22-µm PTFE syringe filters (ChemLand, Stargard, Poland) into glass vials (Witko, Lódź, Poland). The methanol extracts prepared from the commercial and self-cultivated fruiting bodies and the in vitro mycelial cultures were then analyzed. Each sample was analyzed in triplicate. The content of organic compounds was expressed as mg 100 g^−1^ d.w. ± SD.

#### 4.3.2. Analysis of Indole Compounds

The content of indole compounds was determined using HPLC with a UV detector (RP–HPLC–UV). The liquid chromatography equipment used in this study consisted of an HPLC analyzer (Merck Hitachi, Tokyo, Japan) with a constant mobile-phase composition during the analysis (isocratic elution), an L-7400 UV detector (Merck Hitachi, Tokyo, Japan), an L-7100 pump, an L-2350 thermostat, a 4 × 250 mm RP-18 column (Purospher^®^ particle size 5 µm), and a VWR7614 degasser. To determine the content of 5-hydroxy-L-tryptophan (5-HTP) and serotonin, a mixture of 0.1% phosphoric acid and acetonitrile in a 97:3 volume ratio was used as the eluent solution. During the measurement, 20 µL of the test sample was injected. The presence of indole compounds was analyzed at 280 nm and at a flow rate of 1.0 mL min^−1^ for 10 min. In addition, isocratic elution was used to determine other indole compounds such as melatonin, tryptamine, 5-methyltryptamine, and L-tryptophan, using a mixture of methanol, water, and 0.1 M ammonium acetate in a 15:14:1 volume ratio as the eluent solution. The flow rate was set at 1 mL min^−1^, and 20 µL of each sample was injected. The measurement was carried out at 275 nm for 10 min.

#### 4.3.3. Analysis of Sterols

The liquid chromatography equipment used in this study consisted of an HPLC analyzer (Merck Hitachi, Tokyo, Japan), a DAD-L2455 detector, an L-2130 pump, an L-2350 thermostat, an RP-18 4 × 250 mm column (LiChrosfer, grain size 5 µm), and an L-2200 autosampler. Sterol standards were purchased from Fluka, Chemie AG (Buchs, Switzerland). The sterol content was analyzed using gradient elution following the method of Yuan et al. with modifications by Sułkowska-Ziaja et al. [39,40]. Two solvents (A and B) were used as the mobile phase. Solvent A consisted of methanol and water mixed in an 80:20 volume ratio, whereas solvent B consisted of methanol and dichloromethane in a 75:25 volume ratio (the gradient program was the same as previously described) [39]. The flow rate was 1 mL min^−1^, and chromatographic peaks were recorded at 280 nm. The mobile-phase flow rate was 1 mL min^−1^. Twenty microliters of the sample was injected into the column, and analysis was carried out at 280 nm.

#### 4.3.4. Analysis of Lovastatin

The lovastatin content of the samples was determined using the RP–HPLC–UV method, following the method of Pansuriya et al. [41]. The liquid chromatography equipment used in this study consisted of an HPLC analyzer (Merck Hitachi, Tokyo, Japan), which allowed isocratic separation with a constant mobile-phase composition during analysis, an L-7400 UV detector (Merck Hitachi, Tokyo, Japan), an L-7100 pump, an L-2350 thermostat, an RP-18 4 × 250 mm column (Purospher^®^, particle size 5 µm), and a VWR7614 degasser. A mixture of acetonitrile and 0.1% phosphoric acid solution in a 60:40 volume ratio was used as the mobile phase, with a flow rate of 1 mL min^−1^. Twenty microliters of the sample was injected into the column. Detection was carried out at 238 nm, and measurements were carried out over a period of 20 min.

#### 4.3.5. Analysis of Ergothioneine Content

The ergothioneine content in the samples was determined using the RP–HPLC–UV method, following the method of Zhou et al. [42]. The liquid chromatography equipment used in this study consisted of an HPLC analyzer (Merck Hitachi, Tokyo, Japan), which allowed isocratic separation with a constant mobile-phase composition during analysis, an L-7400 UV detector (Merck Hitachi, Tokyo, Japan), an L-7100 pump, an L-2350 thermostat, an RP-18 4 × 250 mm column (Purospher^®^, particle size 5 µm), and a VWR7614 degasser. A mixture of water and methanol in a 99:1 volume ratio, with the addition of 3.0 g of boric acid, was used as the mobile phase to achieve a pH of 5.0. The flow rate of the mobile phase was set at 0.5 mL min^−1^. Twenty microliters of the sample was injected into the column. Measurements were carried out at 257 nm for 20 min.

#### 4.3.6. Analysis of L-Phenylalanine and Phenolic Compounds

The content of phenolic compounds and phenylalanine was determined using HPLC with a diode array detector (RP–HPLC–DAD), following the method of Ellnain-Wojtaszek et al. [43]. The liquid chromatography equipment used in this study consisted of an HPLC analyzer (Merck Hitachi, Tokyo, Japan), a DAD-L2455 detector, an L-2130 pump, an L-2350 thermostat, a 4 × 250 mm RP-18 column (LiChrosfer, particle size 5 µm), and an L-2200 autosampler. The content of phenolic compounds and phenylalanine was analyzed using gradient elution. Two solvents (A and B) were used as the mobile phase. Solvent A consisted of methanol and 0.5% acetic acid solution in a 1:4 volume ratio, whereas solvent B was methanol (gradient previously described by Kała et al., 2021) [11]. Measurements were carried out at 254 nm.

#### 4.3.7. Analysis of Glucan Content

The β-glucan content was analyzed using an assay kit (Megazyme© Ltd., Bray, Wicklow County, Ireland) in accordance with the manufacturer’s instructions, as previously described [44]. For this analysis, 0.1 g of freeze-dried *H. marmoreus* samples were ground using an analytical mill and then sieved through a 0.5-mm sieve. Then, the material was transferred to plastic tubes, 1.5 mL of 37% HCl was added, and the mixture was heated at 30 °C for 45 min. Then, 10 mL of distilled water was added to each tube and incubated for 2 h in a boiling water bath. After neutralization with 2 M KOH, acetate buffer (pH = 5) was added to the samples to achieve a final volume of 100 mL. For further analysis, 0.1 mL of the solution was taken, exo-1,3-β-glucanase (20 U mL^−1^) and β-glucosidase (20 U mL^−1^) were added, and the mixture was incubated in a water bath for 1 h at 40 °C. Then, 3 mL of GOPOD reagent was added, and the mixture was incubated again at 40°C for 20 min. The α-glucan content was analyzed by mixing dried fungal material (0.1 g) with 2 mL KOH (2 M) for 20 min in an ice water bath. After the addition of 8 mL of acetate buffer (pH = 3.8) and 0.2 mL of amyloglucosidase (1630 U mL^−1^), the samples were incubated in a water bath for 30 min at 40 °C. Then, 0.1 mL of the solution was retrieved and mixed with 0.1 mL of acetate buffer (pH 5.0) and 3 mL of GOPOD reagent. The thus-obtained solution was incubated again at 40 °C for 20 min.

The samples prepared as described above were analyzed using a UV/VIS Helios Beta spectrophotometer (UK) at λ = 510 nm and compared with a blind sample. The β-glucan content was calculated by subtracting the α-glucan content from the total glucan content. In both stages, the total glucan, the α-glucan content, the D-glucose content of oligosaccharides and sucrose, and the free D-glucose content were measured. For the detection of 1,3–1,6-β-glucans, the enzyme assay method was used, which is an efficient method for the quantitative determination of β-glucans with special bonds in yeasts and mushrooms. All glucans were decomposed into glucose monomers and measured photometrically. The standard method error was <5% (Megazyme^©^ International Ireland Ltd., Bray, Ireland, 2013) [44].

### 4.4. Determination of Antioxidant Activity

#### 4.4.1. Determination of Antioxidant Activity Using the Dpph^•^ Method

The antioxidant activity was evaluated following the method of Molyneux using the DPPH radical (2,2-diphenyl-1-picrylhydrazyl) [45]. In this method, 0.1 mL of the prepared mushroom supernatant was dissolved in 0.4 mL of distilled water and mixed. Then, 0.1 mL of the resulting solution was mixed with 4.9 mL of 0.1 mM DPPH^•^ dissolved in methanol. The reaction mixture was shaken and then incubated in the dark at room temperature for 30 min. The absorbance of the reaction mixture was measured at 517 nm against the blank using a UV/VIS Helios Beta spectrophotometer (UK). The antioxidant activity was calculated using the following equation: DPPH [%] = [(A0 − A1)/A0] × 100, where A0 and A1 are the absorbances of the reference and test solutions, respectively. The total antioxidant activity was calculated based on the calibration curve of Trolox and expressed as mg Trolox equivalents (TE) per 1 g of dry mass of *H. marmoreus*.

#### 4.4.2. Determination of Antioxidant Activity Using the FRAP Method

The antioxidant activity was also evaluated using the method of Benzie and Strain [46]. In this method, an FRAP reagent (pH = 3.6) was prepared by mixing 2.5 mL of 10 mmol L^−1^ 2,4,6-tripyridy-s-triazine solution with 40 mmol L^−1^ HCl, 2.5 mL of 20 mmol L^−1^ FeCl_3_, and 25 mL of 0.3 mol L^−1^ acetate buffer. Of the prepared mushroom supernatant, 0.1 mL was dissolved in 0.4 mL H_2_O (distilled) and mixed. Then, 0.1 mL of the resulting solution was mixed with 1.9 mL of the FRAP reagent, and the absorbance of the reaction mixture was measured at 593 nm using a UV/VIS Helios Beta spectrophotometer (UK). To prepare the calibration curve, methanol solutions with known Fe(II) concentrations were used. The FRAP results were expressed as M Fe^2+^ per 1 g of dry mass of *H. marmoreus*.

#### 4.4.3. Determination of Antioxidant Activity Using the ABTS Method

To determine the antioxidant activity using ABTS assays, the method of Thaipong et al. (2006) was used with some modifications [47]. The stock solutions used were 7.4 mM ABTS^+^ solution and 2.6 mM potassium persulfate solution. The working solution was prepared by mixing the two stock solutions in an equal volume ratio and allowing them to react for 24 h at room temperature in the dark. Then, the solution was diluted by mixing the ABTS^+^ solution with methanol to obtain an absorbance of 1.1 units at 734 nm, which was measured using a UV/VIS Helios Beta spectrophotometer (UK). For each assay, a fresh ABTS^•+^ solution was prepared. Mushroom extracts were diluted using methanol in a 1:5 volume ratio, and then 0.1 mL of the diluted extracts was allowed to react with 2.9 mL of the ABTS^•+^ solution for 2 h in the dark. The absorbance was read at 734 nm using a UV/VIS Helios Beta spectrophotometer (UK). The standard curve was linear between 25 and 1000 mM Trolox. Results were expressed as mg TE per 1 g of dry mass of *H. marmoreus* [47].

### 4.5. Statistical Analysis

Each test material was analyzed in several independent replicates, and assay results were presented as mean values with SD. Statistical analysis was carried out using the Statgraphics Centurion software. One-way ANOVA with Tukey post-hoc tests was used to analyze the differences in compound concentrations using GraphPad Prism 7.0 at alpha < 0.05.

## 5. Conclusions

This study analyzed solid, liquid (shaken), and liquid (aerated) in vitro cultures of *H. marmoreus* established in 10-L bioreactors, as well as *H. marmoreus* fruiting bodies, to examine their potential roles in improving human health. In addition, for the first time, *H. marmoreus* enriched in zinc and magnesium compounds was obtained, which influenced the metabolism of organic compounds and determined their variable contents. This enrichment increased the contents of Na, Ca, Zn, melatonin, protocatechuic acid, sterols, and total glucans in the mycelial cultures. In contrast, it reduced the contents of K, Cu, 5-HTP, lovastatin, and L-phenylalanine. One of the important findings of this study is the determination of up to six nonhallucinogenic indole compounds, with quantitative results obtained for L-tryptophan, 5-HTP, melatonin, tryptamine, and 5-methyltryptamine, highlighting the potential antidepressant activity of *H. marmoreus*. Furthermore, *H. marmoreus* has been reported as a good dietary source of Fe, Zn, K, Mg, sterols, lovastatin, and glucans, with antioxidant potential. Extremely important is the industrial aspect of the research carried out, because conducting comparative studies of self-cultivated fruiting bodies and those imported from Asia creates opportunities for the introduction of *H. marmoreus* species to the domestic market. The possibility of obtaining *H. marmoreus* species rich in specific substances may also indicate a pharmaceutical application for this species in the future.

## Figures and Tables

**Table 1:** Content of bioelements in *Hypsizygus marmoreus* species [mg 100 g^−1^ d.w. ± SD].

Mushroom Material								
Analyzed Bioelements	F.b. White (Commercial Cultivation)	F.b. Brown (Commercial Cultivation)	F.b. White (Own Cultivation)	F.b. Brown (Own Cultivation)	M.c. White	M.c. Brown	M.c. White + Mg and Zn	M.c. Brown + Mg and Zn
Na	14.5 ± 0.7 ^A^	27.6 ± 0.7 ^B^	25.1 ± 1.4 ^B^	37.1 ± 1.0 ^C^	4.17 ± 0.20 ^D^	6.76 ± 0.31 ^D^	28.5 ± 1.7 ^E^	30.3 ± 0.4 ^E^
K	1211 ± 33 ^A^	1610 ± 61 ^B^	2159 ± 111 ^C^	2656 ± 64 ^D^	686 ± 24 ^E^	788 ± 50 ^E,F^	564 ± 28 ^E,G^	607 ± 41 ^E,G^
Mg	197 ± 9 ^A^	238 ± 4 ^B^	172 ± 9 ^A^	218 ± 5 ^B^	285 ± 8 ^C^	325 ± 16 ^D^	303 ± 11 ^C,D^	330 ± 20 ^D^
Ca	4.10 ± 0.30 ^A^	5.87 ± 0.18 ^B^	8.37 ± 0.31 ^C^	9.51 ± 0.24 ^C^	5.23 ± 0.21 ^B^	6.60 ± 0.37 ^B^	25.8 ± 1.3 ^D^	25.7 ± 0.2 ^D^
Zn	11.2 ± 0.5 ^A,C^	13.4 ± 0.2 ^A,B^	6.47 ± 0.22 ^D^	9.52 ± 0.18 ^C^	12.8 ± 0.3 ^B^	17.4 ± 0.3 ^E^	29.1 ± 1.7 ^F^	30.2 ± 2.2 ^F^
Cu	1.73 ± 0.13 ^A^	1.84 ± 0.09 ^A^	1.14 ± 0.08 ^B^	1.42 ± 0.07 ^C^	2.71 ± 0.10 ^D^	3.53 ± 0.11 ^E^	0.321 ± 0.025 ^F^	0.285 ± 0.045 ^F^
Fe	5.66 ± 0.18 ^A^	7.79 ± 0.55 ^B^	4.48 ± 0.19 ^C^	6.46 ± 0.17 ^A,D^	6.49 ± 0.31 ^A,E^	9.08 ± 0.41 ^F^	8.49 ± 0.10 ^B,F^	7.17 ± 0.25 ^B,D,F^

*n* = 9; d.w.—dry weight; F.b.—fruiting bodies; M.c.—in vitro mycelial cultures; Mg and Zn—mycelial medium additives in the form of MgSO_4_ and ZnSO_4_; the letters next to values represent Tukey’s HSD post-hoc results (*p* < 0.05) according to the compact letter display convention, i.e., a letter refers to the group (A–G) in which there was no significant difference.

**Table 2:** Chemical compounds in the analyzed *Hypsizygus marmoreus* samples.

Mushroom Material								
Analyzed Compounds	F.b. White (Commercial Cultivation)	F.b. Brown (Commercial Cultivation)	F.b. White (Own Cultivation)	F.b. Brown (Own Cultivation)	M.c. White	M.c. Brown	M.c. White + Mg and Zn	M.c. Brown + Mg and Zn
Indole compounds [mg 100 g^−1^ d.w. ± SD]								
L-Tryptophan	59.9 ± 0.5 ^A^	72.1 ± 0.4 ^B^	12.5 ± 0.2 ^C^	8.97 ± 0.38 ^D^	18.8 ± 0.8 ^E^	25.6 ± 1.1 ^F^	17.3 ± 0.4 ^E,G^	18.7 ± 0.2 ^E,G^
5-Hydroxy-L-tryptophan	nd	nd	nd	nd	3.07 ± 0.09 ^A^	11.4 ± 0.8 ^B^	nd	0.319 ± 0.008 ^C^
Serotonin	nd	*	nd	nd	nd	nd	*	nd
Tryptamine	14.2 ± 0.7 ^A^	29.3 ± 0.6 ^B^	*	nd	0.153 ± 0.009 ^C^	nd	*	0.131 ± 0.095 ^C^
5-Methyltryptamine	nd	27.2 ± 0.3 ^A^	nd	nd	3.39 ± 0.02 ^B^	4.71 ± 0.03 ^C^	9.42 ± 0.44 ^D^	3.19 ± 0.01 ^B^
Melatonin	*	nd	*	*	nd	*	4.37 ± 0.15 ^A^	4.99 ± 0.24 ^B^
Sterols [mg 100 g^−1^ d.w. ± SD]								
Ergosterol	74.8 ± 1.0 ^A^	116 ± 1 ^B^	45.3 ± 0.1 ^C^	69.3 ± 0.2 ^D^	24.9 ± 0.1 ^E^	58.8 ± 0.7 ^F^	142 ± 4 ^G^	166 ± 1 ^H^
Ergosterol peroxide	2.60 ± 0.01 ^A^	9.74 ± 0.04 ^B^	nd	*	*	*	6.01 ± 0.21 ^C^	15.8 ± 0.3 ^D^
Phenolic compounds [mg 100 g^−1^ d.w. ± SD]								
*p*-Hydroxybenzoic acid	23.5 ± 0.1 ^A^	10.7 ± 0.1 ^B^	0.300 ± 0.037 ^C^	0.309 ± 0.005 ^C^	nd	1.45 ± 0.03 ^D^	nd	nd
Protocatechuic acid	16.6 ± 0.1 ^A^	11.0 ± 0.1 ^B^	1.65 ± 0.03 ^C^	1.22 ± 0.04 ^D^	3.70 ± 0.11 ^E^	nd	4.14 ± 0.05 ^F^	4.39 ± 0.05 ^G^
Vanillic acid	4.21 ± 0.10 ^A^	nd	nd	nd	nd	nd	nd	nd
Cinnamic acid	6.50 ± 0.03 ^A^	8.88 ± 0.01 ^B^	nd	nd	nd	1.26 ± 0.08 ^C^	nd	nd
Other bioactive compounds [mg 100 g^−1^ d.w. ± SD]								
Lovastatin	74.5 ± 0.6 ^A^	62.4 ± 0.5 ^B^	43.9 ± 0.6 ^C^	66.7 ± 2.6 ^D^	29.2 ± 0.6 ^E^	19.2 ± 0.1 ^F^	15.3 ± 0.1 ^G^	15.0 ± 0.3 ^G^
Ergothioneine	23.4 ± 2.5 ^A^	19.9 ± 1.4 ^A^	17.1 ± 1.4 ^A^	14.7 ± 1.5 ^A^	25.6 ± 1.6 ^B^	73.0 ± 7.4 ^C^	80.4 ± 4.3 ^C^	24.4 ± 1.3 ^A^
L-Phenylalanine	292 ± 16 ^A^	422 ± 12 ^B^	97 ± 2 ^C^	109 ± 2 ^C, D^	132 ± 2 ^D^	228 ± 12 ^E^	111 ± 6 ^D^	103 ± 4 ^C,D^
Glucans [g 100^−1^ g d.w. ± SD]								
Total glucans	41.1 ± 1.0 ^A^	32.0 ± 1.0 ^B^	61.4 ± 1.0 ^C^	58.0 ± 1.5 ^D^	36.0 ± 1.2 ^E^	35.0 ± 0.5 ^E^	41.4 ± 0.8 ^C,F^	42.3 ± 1.2 ^A,F^
α-Glucans **	1.93 ± 0.16 ^A^	3.80 ± 0.11 ^B^	13.2 ± 0.2 ^C^	11.3 ± 0.2 ^D^	10.4 ± 0.4 ^E^	11.6 ± 0.3 ^D^	9.50 ± 0.11 ^F^	9.57 ± 0.37 ^F^
β-Glucans ***	39.1 ± 1.0 ^A^	28.2 ± 1.1 ^B^	48.2 ± 0.8 ^C^	46.8 ± 1.4 ^C^	25.4 ± 1.1 ^B,D^	23.4 ± 0.7 ^D^	31.9 ± 0.8 ^E^	32.7 ± 1.6 ^E^

*n* = 3; d.w.—dry weight; *—trace amount; nd—not detected; F.b.—fruiting bodies; M.c.—in vitro mycelial cultures; Mg and Zn—mycelial medium additives in the form of MgSO_4_ and ZnSO_4_; Total glucans—α-glucans + β-glucans + D-glucose in oligosaccharides, sucrose and free D-glucose; **—α-glucans + D-glucose in sucrose and free D-glucose; ***—β-glucans, calculated by the difference between total glucans with D-glucose in oligosaccharides, sucrose and free D-glucose and α-glucans, including D-glucose in sucrose and free D-glucose (calculations were performed according to the manufacturer’s instructions); the letters next to values represent Tukey’s HSD post-hoc results (*p* < 0.05) according to the compact letter display convention, i.e., a letter refers to the group (A–H) in which there was no significant difference.

## Data Availability

Not applicable.

## Sample Availability

Samples of the compounds are available from the authors.
